# IgA Nephropathy: Current Treatment and New Insights

**DOI:** 10.3390/antib12020040

**Published:** 2023-06-19

**Authors:** Dimitra Petrou, Petros Kalogeropoulos, George Liapis, Sophia Lionaki

**Affiliations:** 1 Department of Nephrology, Second Department of Propaedeutic Internal Medicine, Attikon University Hospital, Medical School, National and Kapodistrian University of Athens, 12462 Athens, Greece; dimitra.petrou90@gmail.com; 2 Department of Pathology, Medical School, National and Kapodistrian University of Athens, 11527 Athens, Greece

**Keywords:** IgA nephropathy, BAFF, APRIL, TRF-budesonide, complement inhibition, SGLT2 inhibitor

## Abstract

IgA Nephropathy (IgAN) is the most common cause of primary glomerulonephritis worldwide. Despite the histopathologic hallmark of mesangial IgA deposition, IgAN is a heterogenous autoimmune disease not only in terms of clinical presentation but also in long-term disease progression. The pathogenesis of the disease is complex and includes the generation of circulating IgA immune complexes with chemical and biological characteristics that favor mesangial deposition and reaction to mesangial under-glycosylated IgA1 accumulation, which leads to tissue injury with glomerulosclerosis and interstitial fibrosis. Patients with proteinuria over 1 g, hypertension, and impaired renal function at diagnosis are considered to be at high risk for disease progression and end-stage kidney disease (ESKD). Glucocorticoids have been the mainstay of treatment for these patients for years, but without long-term benefit for renal function and accompanied by several adverse events. A better understanding of the pathophysiology of IgAN in recent years has led to the development of several new therapeutic agents. In this review, we summarize the current therapeutic approach for patients with IgAN as well as all novel investigational agents.

## 1. Introduction

IgA nephropathy (IgAN) is the most common cause of primary glomerulonephritis worldwide. The diagnostic hallmark of IgAN is the presence of mesangial depositions of IgA, either predominant or codominant with IgG and/or IgM, which are demonstrated by immunofluorescence microscopy [1]. Despite this histopathologic hallmark, IgAN is a heterogenous disease not only in terms of epidemiology or clinical presentation but also in long-term renal disease progression and outcome [1].

The true prevalence of IgAN is unknown since a kidney biopsy is necessary to establish the diagnosis. As a result, variations in disease prevalence may reflect regional differences in screening for kidney disease and other socioeconomic factors. An estimated incidence of biopsy-proven IgAN in the USA is about 1 case per 100,000 persons [2]. It varies among different racial groups, being more common among East Asian individuals, followed by Caucasians, and only rarely affecting African individuals [3]. Although both sexes are equally affected in East Asia, male predominance is documented in North America and Europe. A peak incidence is documented during the second and third decades of life [4].

Clinical presentation of patients with IgAN may vary from asymptomatic microscopic hematuria to macroscopic hematuria with or without proteinuria, acute kidney injury, nephrotic syndrome, or rapidly progressive glomerulonephritis [3]. Approximately half of the patients with IgAN present with one or more recurrent episodes of macroscopic hematuria, often accompanying an upper respiratory or gastrointestinal infection [5]. An initial episode of gross hematuria at age 40 years or older is rarely due to IgAN, and other diagnoses must be ruled out. Incidental detection of asymptomatic microscopic hematuria with or without mild proteinuria during routine assessment applies to almost one-third of patients with IgAN. In these cases, a thorough assessment of urine sediment is of the essence in order to establish the diagnosis of glomerular origin hematuria. Nephrotic syndrome is present in less than 10 percent of patients with IgAN. Acute kidney injury (AKI) or rapidly progressive glomerulonephritis (RPGN) is even rarer and may be due to crescentic IgAN or tubular occlusion because of heavy glomerular hematuria [6]. As a result, IgAN should be always suspected in every individual present with one or more episodes of gross hematuria, especially if accompanied by a recent upper respiratory infection, persistent microscopic hematuria, or even slowly progressive kidney function impairment in the absence of other profound etiology.

Mesangial deposition of IgA is the cornerstone in the pathogenesis of IgAN. IgA is predominantly polymeric IgA of the IgA1 subclass (polymeric IgA1). The factors that lead to the development of disease are still poorly understood. Dysregulated synthesis and metabolism of IgA (resulting in IgA immune complexes with characteristics that favor mesangial deposition) and the mesangial cell reaction to mesangial IgA accumulation are thought to be part of the pathogenetic puzzle. IgAN is an autoimmune disease resulting from the dysregulation of mucosal-type IgA immune responses. The autoantigens are a specific set of IgA1 O-glycoforms displaying poor O-linked galactosylation of the IgA1 hinge region, which result in the generation of hinge glycan-specific IgA and immunoglobulin G (IgG) autoantibodies in susceptible individuals. As a result, some triggers such as mucosal infection or food antigens may drive the production and release of pathogenic IgA into the circulation. There it has the propensity to deposit within the mesangium and trigger glomerular injury [7]. Although IgAN’s pathogenicity is not fully understood, mucosal biopsies from patients with IgA show significantly reduced numbers of polymeric IgA-secreting plasma cells when compared to healthy individuals. Another theory suggests that mesangial IgA is derived from systemically located plasma cells in bone marrow sites. Cytomegalovirus, Hemophilus parainfluenza, Staphylococcus aureus, Streptococcus, toxoplasmosis, and SARS-CoV-2 have been also implicated [8,9,10]. Genetic factors influence the pathogenesis of IgAN as well. A genetic predisposition of this heterogenous disease has been suggested since its presence among certain families has been well described. It is presumed that it does not have classic Mendelian inheritance attributable to a single gene locus but serves as a complex polygenic heterogenous disease. Mucosal tissue (especially in the gastrointestinal tract) constitutes a physical barrier against invaders. The host’s immune system, microbiota, and pathogens are the three main players. With technological advances in genotyping, genetic studies, in particular hypothesis-free genome-wide association studies (GWASs), have documented significant associations of IgAN with several single-nucleotide polymorphisms within or near immune-related genes such as major histocompatibility complex (MHC) loci, thereby highlighting the immune component of this disease. MCH region is critical for antigen presentation and adaptive immunity and could also serve as a potential treatment target [11]. The exact pathogenesis of IgAN remains unclear since genetic, environmental and autoimmune pathways interact with each other. The current model of its pathogenicity involves a four-hit theory that drives disease development and progression. “First hit” refers to the production of aberrant galactose-deficient IgA1 (Gd-IgA1) by plasma cells, leading to the synthesis of autoantibodies directed against the aberrant, Gd-IgA1 (“second hit”). “Third hit” refers to the formation of pathogenic immune complexes circulating in the bloodstream after the binding of autoantibodies to the Gd-IgA1. As a result, circulating immune complexes deposit at the points of filtration (mesangial cells located between the glomerular basement membrane and fenestrated endothelium of the kidney, leading to deposition of local immune activation, inflammation, and glomerular injury (“fourth hit”) [7].

**Figure 1:**
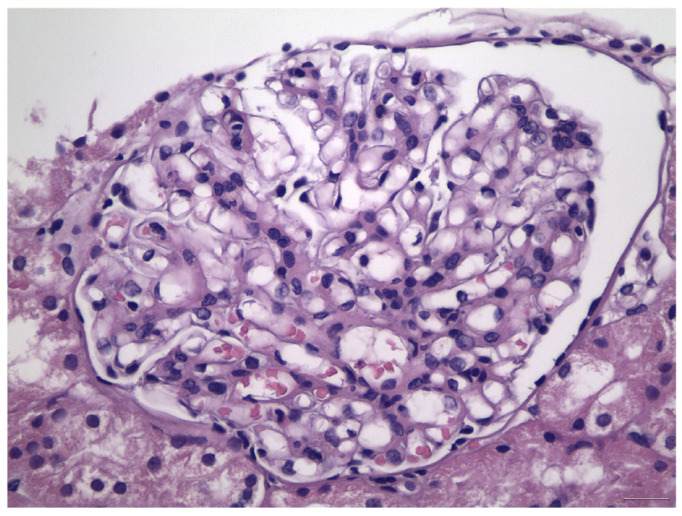
Mild-to-moderate increase in mesangial matrix and mesangial cell proliferation in the case of IgA nephropathy, class M1E0S0T0, according to Oxford Classification.

**Figure 2:**
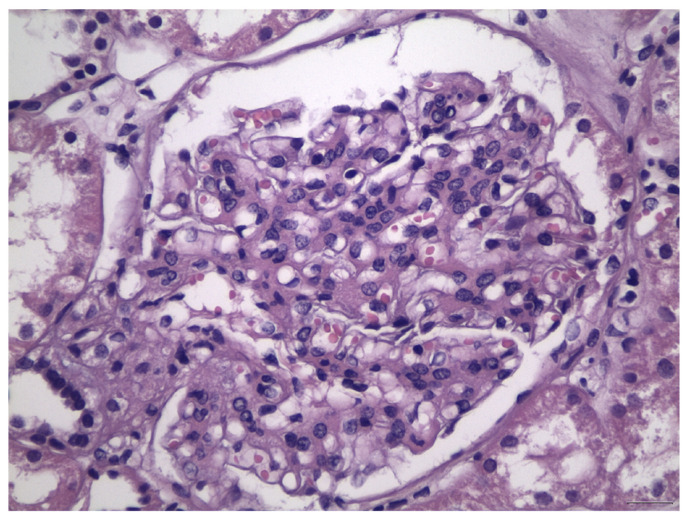
Severe increase in mesangial matrix and mesangial cell proliferation, in association with foci of endocapillary proliferation, in the case of IgA nephropathy, class M1E1S1T1, according to Oxford Classification.

**Figure 3:**
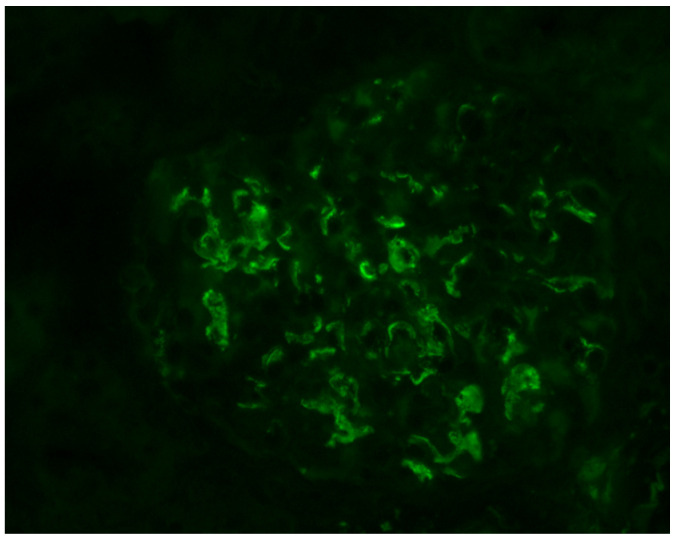
Characteristic, mainly mesangial IgA immunoglobulin deposits, in immunofluorescence examination (IgA ×400).

**Figure 4:**
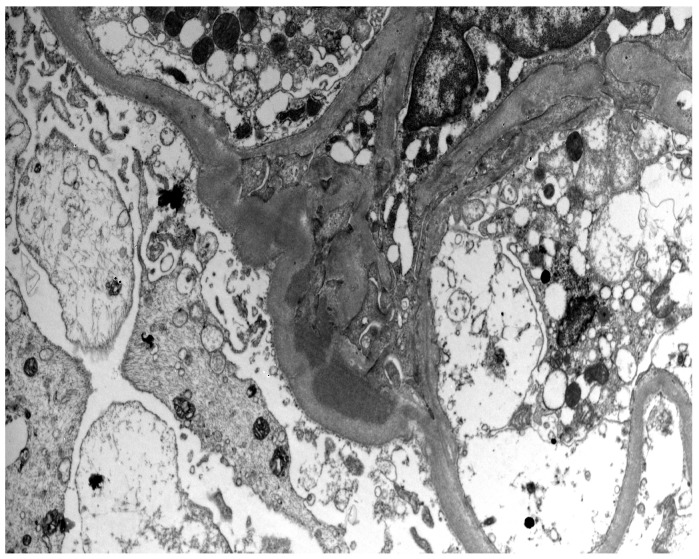
Typical electron-dense deposits in the mesangium, especially beneath the paramesangial basement membrane, in the case of IgA nephropathy (Uranyl acetate and lead citrate ×5600).

Histologically, IgAN is characterized by increase of the mesangial matrix and mesangial cell proliferation, as well as strong, dominant IgA deposition (≥2+), usually accompanied by C3 complement component, and/or IgG immunoglobulin in a lesser degree than IgA immunoglobulin and l light chain deposition, in Immunofluorescence examination. Although many cases show only mesangial proliferation, there are some cases that exhibited both mesangial and endocapillary proliferation with the influx of inflammatory cells into capillary lumens, or even extra-capillary proliferation, with glomerular crescents formation. Segmental glomerular sclerosis/scarring is also another relatively common feature, mimicking sometimes, focal segmental glomerulosclerosis, in cases showing signs of chronicity. Mesangial electron-dense deposits and a few small scattered subendothelial deposits are typically found in Electron Microscopy (EM) examination, although, in some cases, subendothelial deposits can be large. Rarely, a few small subepithelial deposits can also be recognized, although with no true membranous pattern. The tubulointerstitial department may show varying degrees of interstitial fibrosis or tubular atrophy, as well as red blood cell casts into tubular lumens. Thus, IgA nephropathy exhibits a wide spectrum of histological variability, ranging from no essential histological abnormalities to diffuse proliferative and crescentic glomerulonephritis, although most common histological patterns include focal or diffuse mesangial proliferative glomerulonephritis. Without treatment, some of the cases will progress to interstitial fibrosis and tubular atrophy, as well as glomerular scarring and loss, leading to end-stage renal disease. Taking into account the high histological diversity of the disease, but also clinical course variability, several histological grading schemes have been developed and proposed after Berger’s original disease description [12], in order to optimize therapeutic intervention and even to predict patients’ clinical course, among them, the Haas grade scheme is one of the most widely used [13]. The Oxford Classification grading scheme gained interest in the recent years and after Consensus meetings, the MEST-C score is highly recommended to be applied in every case [14,15]. In MEST-C (from Heptinstall’s Pathology of the kidney [16]), M0 or M1 indicate mesangial hypercellularity (≥4 cells in one or more mesangial areas) in ≤50% vs. >50% of glomeruli. E0 or E1 indicate endocapillary hypercellularity in zero vs. one or more glomeruli. S0 or S1 indicate segmental sclerosis in zero vs. one or more glomeruli. T0, T1, or T2 indicate tubular atrophy/interstitial fibrosis in ≤25%, 26% to 50%, or 50% of the renal cortex, respectively. C0 or C1 or C2, if cellular and/or fibrocellular crescents are absent, present in at least one glomerulus or at least 25 percent of glomeruli. Fibrous crescents are not counted toward this score (Figure 1, Figure 2, Figure 3 and Figure 4).

Diagnosis is usually straightforward due to characteristic Immunofluorescence findings, but in some cases, diagnosis can be challenged and obscured. Differential diagnosis includes other diseases that may be IgA dominant in immunofluorescence examination, such as IgA-dominant post-infectious glomerulonephritis (staphylococcal -associated) and Henoch–Schoenlein purpura/IgA vasculitis. The last condition usually presents with systemic involvement (skin, gastrointestinal involvement) and may show vasculitic histological features, such as fibrinoid necrosis and/or crescent formation, although, reliable distinction from IgAN is not possible based only on histological grounds and clinical correlation is recommended in these cases. In post-staphylococcal associated, IgA dominant glomerulonephritis, Electron Microscopy (EM) will reveal large subepithelial humps, but some of these cases can mimic IgA vasculitis due to systemic involvement. Because of the extrarenal manifestations of IgA vasculitis, other systemic diseases may enter also in the differential diagnosis, such as ANCA-associated vasculitis, SLE, and cryoglobulinemia. Serology for ANCA antibodies, lupus-related antibodies and detection of cryoglobulins will allow the distinction. Furthermore, several cases of ANCA-associated vasculitis with mesangial IgA deposits, have been also described. Typically, crescents and/or fibrinoid necrosis are identified in the histology of these cases, but the distinction between the two conditions is made by the ANCA titers (ANCA are positive by ELISA in ANCA-associated cases). Additionally, these cases usually present mild mesangial hypercellularity, as opposed to true IgA vasculitis, which shows prominent mesangial and endocapillary proliferation. Lupus nephritis is characterized by full house pattern and intense C1q staining in contrast with IgA vasculitis. Furthermore, lupus serology and other clinical manifestation of SLE will allow the distinction. In cryoglobulinemia, typical cryoglobulin structures may be found in EM examination in some instances. Cryoglobulin detection in serum examination is essential in these cases. Finally, there are secondary forms of IgA nephropathy, most associated with liver disease and cirrhosis, inflammatory bowel disease, inflammatory arthritis/rheumatologic diseases, dermatologic diseases, and infections, such as HIV.

This article reviewed the current treatment approach for patients with IgAN as well as all treatments that are under development or have recently been approved.

## 2. Risk Stratification for Disease Progression

Prior to treatment commencing, all patients should be assessed for risk stratification on disease progression. Both clinical and histologic features at the time of diagnosis should be taken into consideration. Urine protein excretion above 1 g/day is an important risk factor [17]. Patients excreting less than 1 gr per day have a lower progression rate, in contrast with those excreting more than 3 to 3.5 g/day [18]. However, the magnitude of proteinuria is not a two-way relationship, since some studies suggest that almost up to one-third of patients with high-risk histologic features who eventually experienced reduced kidney function had onset proteinuria less than 1 g/day [19]. Thus, proteinuria of less than 1 gr per day by itself does not guarantee a good outcome. Barratt et al., however, just published the results of their IgA nephropathy UK cohort, including individuals with IgAN plus proteinuria >0.5 g/day or eGFR < 60 mL/min/1.73 m^2^ with a median follow-up of 5.9 years. Unfortunately, 50% of patients reached kidney failure or died in the study period, indicating that outcomes are generally poor with few patients expected to avoid kidney failure in their lifetime. Significantly, patients traditionally regarded as being “low risk”, with proteinuria <10 mg/mmol, still have high rates of kidney failure within 10 years [20]. Hypertension on diagnosis is also an established risk factor for disease progression or death [17,21]. Impaired kidney function at diagnosis or during the course of the disease is also associated with a worse renal prognosis [18,22]. Hematuria is another important risk factor, since its gross magnitude and persistence are associated with worse kidney prognosis, whereas the resolution of hematuria is associated with better renal prognosis [23,24]. Besides these clinical features, there are also histologic findings on kidney biopsy which have been identified as strong risk factors for disease progression. They include crescent formation, mesangial depositions, or capillary loops, as well as markers of chronic fibrotic damage. They are all described in the revised Oxford classification of the IgAN MEST-C Score, as stated above. The International IgAN Prediction Tool (IIgAN-PT) is available as an online calculator, incorporates clinical and histologic data on biopsy, and serves as a prediction tool for five-year-risk of 50% decline in eGFR or kidney failure [25,26]. It cannot, however, determine the likely impact of any treatment regimen.

## 3. Supportive Care in All Patients—The Role of SGLT2 Inhibitors

Unlike other glomerular diseases, IgAN is primarily treated through non-immunosuppressive therapy. In the absence of variant forms of IgAN (e.g., IgAN with MCD or AKI or RPGN) and secondary causes (e.g., autoimmune disease, liver cirrhosis, inflammatory bowel disease, HIV, hepatitis, IgA vasculitis), optimized supportive care is initially suggested. Optimized supportive care focuses on blood pressure management, reduction of proteinuria, lifestyle modification, and total cardiovascular risk addressing [26]. High-quality data support the benefit of blood pressure (BP) control and reduction of proteinuria to delay kidney disease progression in all chronic kidney disease (CKD) populations [27]. Control of BP involves initially lifestyle modifications, such as salt restriction, dietary modification, weight reduction, smoking cessation, lipid lowering, and physical exercise as part of a holistic approach. If medication is needed in patients with hypertension and IgAN and proteinuria >0.5 g/day, it is recommended that initial therapy be conducted with either an angiotensin-converting enzyme inhibitor (ACEi) or angiotensin II receptor blocker (ARB) [26,28,29,30]. In an RCT with 44 patients with IgAN by Praga et al., ACE inhibitors significantly improved renal survival in proteinuric IgAN with normal or moderately reduced renal function in comparison with alternative antihypertensive agents [28]. It is also recommended that all patients with proteinuria >0.5 g/day, irrespective of whether they have hypertension, should be treated with either an ACEi or an ARB [26]. When commencing RAS blockage in patients with IgAN who are normotensive, low-dose therapy should be initiated. Dose titration until the maximally tolerated dose is recommended in order to achieve a maximal reduction in proteinuria while minimizing side effects, such as orthostatic hypotension.

Sodium-Glucose Cotransporters (SGLTs) are proteins that occur primarily in the kidneys and play an important role in maintaining glucose balance in the blood. SGLT1 and SGLT2 are the two most known SGLTs of this family. Sodium-glucose Cotransporter 2 (SGLT2) is located in the proximal tubule of the nephron and causes dynamic reabsorption of 90% of filtered glucose together with sodium. Sodium-glucose Cotransporter 2 inhibitors are newer oral antidiabetic drugs that successfully inhibit glucose reabsorption and cause glycosuria and natriuresis. Glycosuria results in a modest decrease in plasma glucose levels while natriuresis causes a decrease in preload and plasma volume, with an accompanying modest decrease in blood pressure. In addition, intraglomerular pressure and glomerular hyperfiltration are reduced due to the vasoconstriction of the afferent arteriole through the mechanism of tubuloglomerular negative feedback (TGF). Their last function, combined with the reduction of blood pressure caused by natriuresis, results in their nephroprotective effect [31]. SGLT2 inhibitors are also indicated in maximal supportive care of patients with IgAN and proteinuria. DAPA-CKD trial was initially designed to evaluate the effect of dapagliflozin on renal, cardiovascular outcomes, and mortality in people with CKD, with or without T2D demonstrating profound kidney protective benefits, there are data suggesting that these benefits also extend both to diabetic and nondiabetic proteinuric CKD patients, including 270 individuals with IgAN [32,33]. As an example, in a prespecified analysis of individuals with IgAN in a DAPA-CKD study, the primary composite endpoint (sustained decline in eGFR of ≥50 percent, ESKD, or death from a kidney disease-related or cardiovascular cause) occurred in six patients (4 percent) receiving dapagliflozin compared with 20 (15 percent) receiving placebo, a benefit that was independent of baseline proteinuria (hazard ratio, 0.29; 95% confidence interval, 0.12, 0.73). Dapagliflozin also reduced the urine albumin-to-creatinine ratio by 26 percent relative to the placebo [32]. The EMPA-KIDNEY trial evaluated the use of another SGLT2 inhibitor, empagliflozin, in 6609 patients with CKD who had an estimated glomerular filtration rate (eGFR) of at least 20 but less than 45 mL per minute per 1.73 m^2^ of body-surface area, or who had an eGFR of at least 45 but less than 90 mL per minute per 1.73 m^2^ with a urinary albumin-to-creatinine of at least 200. A total of 817 patients with IgAN have been included. The primary outcome was a composite of progression of kidney disease (defined as end-stage kidney disease, a sustained decrease in eGFR to <10 mL per minute per 1.73 m^2^, a sustained decrease in eGFR of ≥40% from baseline, or death from renal causes) or death from cardiovascular causes. Among a wide range of patients with chronic kidney disease who were at risk for disease progression, empagliflozin therapy led to a lower risk of progression of kidney disease or death from cardiovascular causes than placebo [34].

## 4. Immunosuppressive Therapy in High-Risk Patients

Despite maximal supportive care, there are patients who remain at high risk of progressive CKD. These patients may need immunosuppressive treatment in addition to standard therapy. Multiple studies demonstrate that proteinuria is the most powerful predictor of long-term renal outcome [17,18,19]. Proteinuria reduction <1 g/day is a reasonable treatment target [35]. Improvement of hematuria and stabilization of renal function are also goals of therapy. Patients with IgAN and persistent proteinuria ≥1 g/day, despite three to six months of optimized supportive care, are deemed at high risk of disease progression and may be considered eligible for immunosuppressive therapy. On the other hand, patients with IgAN and proteinuria <1 g/day are considered at lower risk of progressive disease and continue maximal supportive care. However, immunosuppressive therapy comes at a cost of significant risk of treatment-related toxicity. A detailed discussion between caregivers and patients is important in order to discuss possible risks and benefits. Immunosuppressive therapy is contraindicated in patients with evidence of severe and irreversible kidney damage (eGFR < 30 mL/min/1.73 m^2^ for more than 3 months, small echogenic kidneys, interstitial fibrosis, tubular atrophy, or severe glomerulosclerosis on kidney biopsy) since they usually tend to have an increased risk of treatment-emergent toxicity with no benefit.

For patients who are considered to be at high risk of disease progression, glucocorticoid therapy in combination with supportive care is recommended [26]. The initial dose of glucocorticoids is maintained for a minimum of two months, at which time the dose is tapered over four months. The largest available RCT of glucocorticoids is the TESTING study. The trial enrolled 503 patients with IgAN with persistent proteinuria ≥1 g/day despite optimal supportive care for at least three months and aimed to evaluate the efficacy and adverse effects of methylprednisolone in patients with IgAN. The primary endpoint was a composite of a 40% decline in eGFR, kidney failure (dialysis, transplant), or death due to kidney disease. Initially, participants were randomized in a 1:1 ratio to receive either oral methylprednisolone (0.6–0.8 mg/kg/d, maximum 48 mg/d) or placebo. However, because of an interim analysis showing an excess of serious adverse effects (mostly serious infection) in the methylprednisolone arm (14.7 vs. 3.2 percent), the trial protocol was revised to include another arm of 241 additional patients to receive a reduced dose of oral methylprednisolone (0.4 mg/kg/day, maximum dose 32 mg/day) or placebo, with ongoing follow-up of all trial participants, including those in the original high-dose cohort. At a mean of 4.2 years, the primary endpoint occurred in fewer patients in the glucocorticoid group than in the placebo group (29 vs. 43 percent). Although there was no significant difference in deaths due to kidney failure or from any cause between the groups, the risk of ESKD was lower in the glucocorticoid group. However, the incidence of serious adverse events was increased with oral methylprednisolone, mainly with high-dose therapy [35,36,37]. Additionally, the STOP-IgAN study suggested the efficacy of glucocorticoids. It included 162 individuals with IgAN and a high risk of disease progression and suggested a reduction in proteinuria (17% vs. 5%, *p* < 0.001) at 3 years on an early analysis [38]. Long-term data analysis at 7 years, though, revealed that the addition of immunosuppressive therapy to the standard of care did not alter the long-term outcome in terms of eGFR loss, ESKD, or death [39]. As a result, the clinical benefit of glucocorticoids in IgAN is not yet established and should be given with extreme caution or even avoided under certain circumstances, such as severely impaired renal function with an eGFR < 30 mL/min/1.73 m^2^, diabetes, latent infections, active peptic ulceration, severe osteoporosis, or uncontrolled psychiatric illness [26]. A Retrospective Analysis from the VALIGA Trial studied 1147 individuals with IgAN. Overall, 46% of patients received immunosuppression, of which 98% received glucocorticoid. This study supports the use of corticosteroids in addition to renin–angiotensin system blockade with a proteinuria >1 g/day, even with an initial eGFR ≤50 mL/min per 1.73 m^2^ [40]. When immunosuppressive treatment is indicated, patients should also receive prophylaxis against Pneumocystis Jirovecii pneumonia, gastroprotection, and osteoporosis prevention therapy. The treatment regimens used in IgAN are summarized in Table 1.

Several immunosuppressive treatment regimens have been studied but documented no evidence of efficacy in IgAN (cyclophosphamide, azathioprine, calcineurin inhibitors, rituximab) [46]. There are few data to support the efficacy of mycophenolate mofetil (MMF) as a first-line treatment for IgAN in the past [47,48]. In contrast, data supporting the efficacy of MMF come from a recent open-label trial of 170 Chinese patients with IgAN and at high risk for disease progression, who were randomly assigned to MMF (1.5 g/day for 12 months, then tapered to a maintenance dose of 0.75 to 1 g/day for at least six months) plus supportive care or supportive care alone. After a 3-year follow-up, patients receiving MMF had a lower annual decline in GFR (−1.2 vs. −3.8 mL/min/1.73 m^2^), a lower rate of doubling of serum creatinine (7.1% vs. 21.2%), while the rate of ESKD and death from cardiovascular causes did not differ significantly [49].

Targeted-release formulation of budesonide (TRF-budesonide) is an oral targeted-release formulation of the glucocorticoid budesonide that releases the drug in the distal ileum, where most Peyer patches are located. Mucosal B lymphocytes localized within Peyer patches are postulated to be a source to produce poorly galactosylated immunoglobulin A1 (IgA1). The safety and efficacy of TRF-budesonide were evaluated in a randomized, placebo-controlled phase 3 trial (NefIgArd) of 199 patients with IgAN. In Part A, patients were treated with TRF-budesonide or a placebo for nine months. At nine months, the 24-h urine protein-to-creatinine ratio was 27% lower in the TRF-budesonide arm, along with a benefit in eGFR decline (3.87 mL/min/m^2^ vs. placebo). Rates of adverse events, including infections were similar between the two groups, but the TRF-budesonide group was more prone to discontinuation of treatment. Hypertension, peripheral and facial edema, muscle spasms, and acne were more frequent in the TRF-budesonide arm, probably due to the systemic glucocorticoid effect. Based on the results of this study, TRF-budesonide was granted accelerated approval by the United States Food and Drug Administration (FDA) for the reduction of proteinuria in patients with IgAN at risk of rapid disease progression (defined as UPCR ≥ 1.5 g/g; proteinuria > 2 g/day). Long-term efficacy and safety data will be reported in Part B of this trial which will include 365 patients [50]. Other preparations of enteric budesonide have also reduced proteinuria in uncontrolled studies [51,52].

## 5. Other Investigational Agents

### 5.1. Inhibition of Immune Complex-Activated Complement Activity

Topical complement activation has a role in the pathogenesis of IgAN, as pathogenic immune complexes of galactose deficient IgA1 can activate both the alternative and lectin pathways, leading to the generation of the membrane attack complex C5b-9, inducing mesangial cell apoptosis and glomerular inflammation via IL-6 and TGF-β1 production [53,54,55]. Glomerular C3 deposition in IgAN predicts more severe clinical features, worsen histopathological characteristics, and long-term poor renal survival [54,56,57]. Several phase II/III studies are already underway, targeting various key points in the activation of the complement cascade. Factors under investigation that target the inhibition of C5a activation are avacopan, an anti-C5a receptor antagonist, which showed an improvement in the slope of the UPCR in a short-term pilot study (NCT02384317) [58], ravulizumab, an eculizumab-derived long-acting C5-blocking antibody (SANCTUARY study, NCT04564339), and cemdisiran, a small interfering RNA-targeting C5 (NCT03841448). In addition, agents that inhibit the complement activation pathway are being tested in phase ΙΙ trials such as APL-2 (NCT03453619) and iptacopan (NCT03373461). Iptacopan was well tolerated and led to a continuous reduction in proteinuria at 6 months and will be further evaluated in the ongoing Phase ΙΙΙ APPLAUSE-IgAN trial (NCT04578834) [59]. IONIS-FB-LRx is an antisense inhibitor of complement factor B messenger ribonucleic acid (CFB mRNA), which is under phase II clinical trial and the result is pending (NCT04014335). Finally, Narsoplimab, which is a human monoclonal antibody against mannan-associated lectin-binding serine protease-2 (MASP-2), inhibits lectin complement pathway activation. Interim analysis from a phase II clinical trial suggests that narsoplimab treatment reduced proteinuria and preserved kidney function [60]. The safety and efficacy of narsoplimab in IgAN patients with more than 1 g/d proteinuria are currently being assessed in the phase III ARTEMIS-IGAN study (NCT03608033).

### 5.2. Inhibition of BAFF/APRIL Signaling

BAFF (B-cell activating factor) and APRIL (a proliferation-inducing ligand) are tumor necrosis factor family ligands involved in B cell and plasma cell function and survival and the pathogenesis of several autoimmune diseases, including IgAN [61]. In patients with IgAN, there is increased expression of APRIL, which is associated with increased expression of Gd-IgA1 antibodies [62]. Thus, targeting APRIL and BAFF may reduce Gd-IgA1 antibody levels. The phase II/III BRIGHT-SC study (NCT02062684), which studied blisibimod, a monoclonal antibody against both soluble and membrane BAFF, showed a reduction of proteinuria compared to placebo. Anti-APRIL antibodies sibeprenlimab (NCT05248646) and BION-1301 (NCT03945318) are in phase III and II clinical trials, respectively, to test efficacy and safety in patients with IgAN [63]. Atacicept, a soluble TACI-Immunoglobin fusion protein, which targets both BAFF and APRIL, showed a reduction in Gd-IgA1 antibody levels and proteinuria when evaluated in the randomized phase II JANUS study (NCT02808429) in 16 patients with IgAN [64]. Further evaluation of safety and efficacy is underway in the ORIGIN phase IIb clinical study (NCT04716231). Finally, telitacicept, another BAFF/APRIL inhibitor, showed proteinuria reduction in 44 patients with IgAN in a phase II clinical trial (NCT04905212) [65].

### 5.3. Plasma Cell and B Cell Depletion

Targeting of Gd-IgA1-producing immune cells could improve renal outcomes in patients with IgAN. Rituximab, a chimeric monoclonal antibody targeted against CD20, shows lack of efficacy in a randomized, controlled clinical trial [66]. The promising felzartamab, a fully human IgG1 monoclonal antibody designed to deplete CD38+ plasma cells, is in a phase II clinical trial for patients with IgAN and an increased risk of disease progression (NCT05065970). Finally, bortezomib, a proteasome inhibitor that depletes plasma cells and is approved for the treatment of multiple myeloma, showed complete remission of proteinuria in 4 out of 8 patients in the first year of follow-up in a pilot trial (NCT01103778) [67].

### 5.4. Inhibition of Endothelin A Receptor and Angiotensin II Subtype 1 Receptor

Endothelin-1 (ET-1), largely through activation of endothelin A receptors, and angiotensin II have a role in kidney function decline by contributing to inflammation and fibrosis in the kidney, changes to the shape of podocytes, podocyte loss, mesangial cell proliferation and increased permeability of the glomerular filtration barrier. Both are also causing vasoconstriction, leading to increase glomerular pressure [68,69]. Sparsentan is a dual-acting antagonist of both endothelin type A (ETA) and angiotensin II subtype 1 (AT1) receptors. The PROTECT trial (NCT03762850) is a phase ΙΙΙ, multicenter, international, randomized, double-blind, parallel-group, active-controlled study, which compares the safety and efficacy of sparsentan vs. irbesartan in adults with biopsy-proven IgAN. The estimated completion date of the study is July 2026. Atrasentan is a potent and selective endothelin A receptor antagonist with the potential to provide benefits in IgA nephropathy and other proteinuric glomerular diseases by reducing proteinuria. The ALIGN Study is a phase 3, double-blind, placebo-controlled study to compare the efficacy and safety of atrasentan to placebo in patients with biopsy-proven IgAΝ at risk of progressive loss of renal function. The estimated completion date of the study is 1 December 2025.

All the investigation agents are summarized in Table 2.

## 6. Conclusions

IgAN is the most common primary glomerulonephritis worldwide and had different heterogeneity in terms of clinical presentation and risk of progression. The current therapeutic options in patients who are at increased risk for ESKD are mainly glucocorticoids, which, however, do not appear to be of long-term benefit and are associated with the appearance of several adverse events. In terms of supportive care, SGLT2 inhibitors show that they can reduce proteinuria, with more studies needed to understand their effect on long-term outcomes. A FIND-CKD trial is also underway to evaluate the effect of finerenone, a novel non-steroidal mineralocorticoid receptor antagonist (MRA) in the non-diabetic CKD patient population (NCT05047263). The combination treatment with SGLT2i may have further applications in patients with IgAN. Dual-acting inhibitors of ETA and AT1 receptor are also another future option in terms of supportive care. More data are required to identify the role of these inhibitors in combination with SGLT2i as a new possible therapeutic strategy in patients with IgAN. TRF-budesonide is an alternative to glucocorticoids for patients who cannot tolerate a six-month of moderate dose, but there is a lack of studies comparing it with moderate-dose glucocorticoid regimens. As discussed above, agents targeting various disease pathogenesis processes, including regulation of pathogenic IgA1 and immune complexes production and blockade of complement cascades, are being evaluated in ongoing clinical trials worldwide.

## Figures and Tables

**Table 1:** Glucocorticoid regimens used in clinical trials of IgAN.

Study	Medication	Initial Dose	Taper	Total Exposure
TESTING 2022 (Lv) [36]	Methylprednisolone	0.4 mg/kg orally once daily (maximum dose: 32 mg/day) for 2 months	Reduce daily dose by 4 mg every month for 4 months	6 months
TESTING 2017 (Lv) [37]	Methylprednisolone	0.6 to 0.8 mg/kg orally once daily (maximum dose: 48 mg/day) for 2 months	Reduce daily dose by 8 mg every month for 4 months	6 months
Manno et al. [41]	Prednisone	1 mg/kg orally per day (maximum dose: 75 mg/day) for 2 months	Reduce daily dose by 0.2 mg/kg every month for 4 months	6 months
Lv et al. [42]	Prednisone	0.8 to 1 mg/kg orally per day for 2 months	Reduce daily dose by 5 to 10 mg every 2 weeks for 4 months	6 months
Pozzi et al. [43] STOP-IgA (Rauen) [44]	Methylprednisolone (IV) and Prednisolone/prednisone (oral)	Methylprednisolone 1 g IV for 3 days at the start of months 1, 3, and 5 and Prednisolone or prednisone 0.5 mg/kg orally every other day on remaining days for 6 months	None	6 months
NEFIGAN (Fellström) [45]	TRF-budesonide	16 mg orally daily for 9 months	Reduce dose to 8 mg once daily for 2 weeks, then discontinue	9 months

**Table 2:** Investigation agents in clinical development for the treatment of IgAN.

Agent	Phase	Registration No	Mechanism of Action
Inhibition of Immune Complex-Activated Complement Activity			
Avacopan	II	NCT02384317	anti-C5a receptor antagonist
Ravulizumab	II	NCT04564339	long-acting C5-blocking antibody
Cemdisiran	II	NCT03841448	small interfering RNA-targeting C5
APL-2	II	NCT03453619	cyclic peptide inhibitor of C3 and C3b
Iptacopan	III	NCT04578834	small-molecule inhibitor of complement factor B
IONIS-FB-LRx	II	NCT04014335	antisense inhibitor of complement factor B messenger ribonucleic acid
Narsoplimab	III	NCT03608033	human monoclonal antibody against (MASP-2)
Inhibition of BAFF/APRIL Signaling			
Blisibimod	II/III	NCT02062684	monoclonal antibody against both soluble and membrane BAFF
Sibeprenlimab		NCT05248646	monoclonal IgG2κ antibody targeting APRIL
BION-1301	I/II	NCT03945318	monoclonal IgG4 antibody targeting APRIL
Atacicept	IIb	NCT04716231	BAFF/APRIL dual inhibitor
Telitacicept	II	NCT04905212	BAFF/APRIL dual inhibitor
Plasma Cell Depletion			
Felzartamab	II	NCT05065970	monoclonal IgG1 antibody targeting CD38
Bortezomib	NA	NCT05383547	proteasome inhibitor that depletes plasma cells
Inhibition of Endothelin A Receptor and Angiotensin II Subtype 1 Receptor			
Sparsentan	III	NCT03762850	endothelin A receptor and angiotensin II subtype 1 receptor inhibitor
Atrasentan	III	NCT04573478	endothelin A receptor antagonist

## Data Availability

The data that support the findings of this study are available from the corresponding author, upon reasonable request.
